# Effects of Pharmacist-Led Clinical Pathway/Order Sets on Cancer Patients: A Systematic Review

**DOI:** 10.3389/fphar.2021.617678

**Published:** 2021-05-21

**Authors:** Zhiyuan Tan, Zhiheng Yu, Ken Chen, Wei Liu, Rongsheng Zhao

**Affiliations:** ^1^ Department of Pharmacy, Peking University Third Hospital, Beijing, China; ^2^ Department of Pharmacy Administration and Clinical Pharmacy, School of Pharmaceutical Sciences, Peking University, Beijing, China; ^3^ Therapeutic Drug Monitoring and Clinical Toxicology Center of Peking University, Beijing, China

**Keywords:** pharmacist, clinical pathway, order sets, cancer, systematic review

## Abstract

**Background:** Pharmacist-led clinical pathways/order sets (PLCOs) were first applied for designated diseases and surgical operations, such as cancer. They were not used in pharmacotherapy until recently. After screening a large number of publications, we found that PLCOs were rarely accessible.

**Objective:** To evaluate the effects and the changes of relevant medical outcomes of PLCOs.

**Methods:** Articles from PubMed, Embase, Cochrane Library, China National Knowledge Infrastructure, Wanfang database, and China Biology Medicine disc (CBM) were systematically retrieved. Clinical research comparing cancer patients’ clinical effects with or without clinical pathway/order sets was performed. Two reviewers performed quality assessment, and the data were abstracted independently. A narrative synthesis of the extracted data was performed due to heterogeneity.

**Results:** Nine studies were identified, including six uncontrolled before–after studies and three case-series studies. The scopes of PLCOs of included research can be divided into two types, one focusing on chemotherapy agents and the other on the managements of chemotherapy-induced complications. The PLCOs shortened hospital length of stay, decreased initial antibiotic time intervals in patients with febrile neutropenia, reduced medication error incidence, and increased physicians’ adherence rate to clinical pathway/order sets. Moreover, three articles included economic effects showing positive savings on medication costs through PLCOs.

**Conclusion:** PLCOs can have beneficial effects on medication effectiveness, safety, and economic outcomes. Nevertheless, clinical pathway/order sets need to be further optimized and expanded to other clinical areas.

## Introduction

The concept of evidence-based pharmacy practice has been well recognized; however, there is poor adherence by both physicians and patients to the practice (Arts et al., 2016). Clinical pathways (CPs) are patient-centered medical care plan that are based on evidence-based guidelines that are initiated and implemented by healthcare professionals (Mingzi, 2010). The purpose was to decrease heterogeneity in treatment and improve the quality, efficacy, and accuracy of care according to standardized outcome metrics (Sylvester and George, 2014). Similarly, order sets are groups of medical orders that standardize diagnosis and medical treatment following clinical guidelines or consensus (Ahmadian and Khajouei, 2012). An order set allows healthcare professionals to issue prepackaged groups of orders that apply to a specific disease (Haynes et al., 2009). One main impetus for order sets comes from the need to improve user adherence for computer-based physician order entry systems, by decreasing the time physicians need to type orders. Using order sets decreases the time spent on prescribing (Mehta et al., 2018). It is worth mentioning that a similar concept of treatment algorithm (TA) came into prevalence in the U.S. medical care system (DiGioia and Rubash, 1991; Green et al., 2019). These two concepts have differences and complementary areas of practice.

To be more specific, standardized care of cancer patients, which can also be defined as “care pathways,” is needed in both the ward (Dear et al. (2017)) and outpatients (Ekstedt et al. (2019)), due to the complicated processes of chemotherapy and various treatment-related or postoperative complications. Thus, opportunities (Shabaruddin et al. (2010); Busby et al. (2011); Oyebode (2013); Flagg et al. (2013); Kataoka et al. (2017)) have emerged for hematology/oncology pharmacists, to optimize medication procedures (Shipman and Arzola (2010); Al Sudairy et al. (2014)), according to guidelines.

Despite many investments and progress, oncology is still an area with significant unmet medical needs that need new therapies and more optimized current therapies (van Hasselt and van der Graaf, 2015). Global Cancer Statistics 2020 showed that (Sung et al. (2021)) an estimated 19.3 million new cancer cases and 10.0 million cancer deaths occurred in 2020. To be more specific, China ranked the first in both the incidence and mortality, which could be a serious burden in next several decades. Various innovative products have emerged into the clinic in recent years, like targeted small molecules, monoclonal antibodies, PD-1/PD-L1 inhibitors, and CAR T cells (Jain, 2021). Different from medicines for other diseases, the consequences of dosing and administration errors are potentially severe, requiring forcing of rescue medications, adequate hydration, and strict dose range checks in oncology (Chen and Lehmann, 2011). What is more, the healthcare professionals always cannot be familiar with the medication process, which means the CP and order sets are of great significance in oncology.

Both CP and order sets were first applied for designated diseases and surgical operations, and commonly were led by physicians and nurses (Durant, 2017). They were not used for pharmacotherapy until recent years (Best et al., 2011). After screening a large number of publications, we found that Pharmacist-led clinical pathway/order sets (PLCOs) could be found rarely. The term “led” means practice implemented by pharmacists only or a multidisciplinary team led by pharmacists. This article aimed to evaluate the effects of PLCOs on effectiveness, safety, and economic outcomes of cancer patients receiving chemotherapy.

## Methods

A systematic review was conducted following the reporting and methodological standards recommended by Preferred Reporting Items for Systematic Reviews and Meta-Analysis (PRISMA) statements (Liberati et al., 2009).

### Literature Research and Screening

Eligible research or systematic reviews were identified through a systematic search, performed in PubMed, Embase, Cochrane database, CINAHL, and three Chinese databases: China National Knowledge Infrastructure (CNKI), Wanfang database, and China Biology Medicine disc (CBM), from inception to March 2020. This restriction is to ensure that the most recent publications are covered in this review while minimizing the possibility of inadvertently excluding older studies. The terms we used include Neoplas*, Tumor*, Malignancy, Malignancies, Cancer*, Therioma, Lymphoma, Leukemia; Critical Pathway, Critical Paths, Clinical Paths, Clinical Pathways, Treatment algorithm, Medical algorithm, Order template, Order set, electronic prescribing; Pharmaceutic Services, Pharmaceutical Service, Pharmacist intervention, Pharmacy Services, Hospital Pharmaceutical Service, Hospital Pharmaceutic Service, Pharmaceutical care, Pharmacist, clinical pharmacist, Pharmacist-driven. Logical character “OR” was used for intra-link and “AND” for inter-link. The entire search strategy was listed in the Supplementary Material S1. A manual supplemental retrieval of gray literatures and other resources (conference proceedings and dissertations, web search engines, web repositories, and library catalog) were performed. References lists from related reviews and manuscripts were also checked.

### Study Selection (PICOs)

Two authors, a postgraduate student pharmacist specializing in oncology and a hematology/oncology specialist, reviewed titles and abstracts to identify relevant articles. Population included patients who have confirmed diagnosis of all tumor types. Interventions: PLCOs. PLCOs were defined as a standard medication process for specific disease or drug led by pharmacists. Due to the unique characteristic of the study, control groups may be unavailable; thus, it was not suitable for study selection. Outcomes were divided into primary outcomes and secondary outcomes. Primary outcomes were defined as all survival-related endpoints like overall survival (OS), and secondary outcomes were the others like tumor-related hospitalization, laboratory test changes, life quality, and medical expedition. Two reviewers independently assessed the titles and the abstracts of the screened citations. Disagreements between authors about inclusion were resolved through consensus. Study types included (i) study subjects who were inpatients, outpatients, and patients receiving chemotherapy in community with malignant disease; (ii) those that were published (non)/randomized controlled trials (RCTs and non-RCTs), controlled before–after study (CBAs), uncontrolled before–after study (UBAs), case–control, cohort, and case-series study; (iii) studies containing two interventions: first: led by pharmacists and second: applying clinical pathways and order sets; and (iv) study outcomes: effectiveness, safety, and economics. No language limitation was set, and therefore, we additionally searched Chinese literature databases. The exclusion criteria were as follows: (i) duplicate publication, (ii) no clear address or cannot tell whether written by hospital/community pharmacists, (iii) reviews or studies simply introduced CP/order sets without robust data, and (iv) full articles not available.

### Risk of Bias Assessment

The qualities of the included RCTs were assessed with Cochrane Collaboration’s risk-of-bias tool, and the Newcastle–Ottawa Scale (NOS), which addressed representativeness of the exposed cohort, selection of the nonexposed cohort, ascertainment of exposure, and demonstration that the outcome of interest was not present at the start of the study, was applied for assessment of cohort studies, case–control studies, CBAs, and UBAs. Two authors independently assessed the risk of bias and resolved disagreements by discussing with a third author. Each paper indicates that using the NOS checklist for cohort studies, which calculates a score for papers between 0–9, based on the reliability of the reported data. A maximum of nine points was used to appraise each observational study in the following domains: selection and selective reporting (maximum of four points), comparability (maximum of two points), and outcome (maximum of three points).

### Data Extraction

Data were extracted and recorded by one author in consultation with the other authors with the use of a predesigned electronic table with the relevant information. Subjects, intervention type, demography, disease, outcomes, and conclusions were extracted.

### Data Synthesis and Analysis

Meta-analysis was not applicable due to the heterogeneity in study populations, intervention type, and outcome measurements. Most studies had a before–after design that could definitely have high levels of bias.^7^ Consequently, a narrative synthesis of the recorded data was performed. After performing data synthesis and categorizing studies as described above, the final report will be prepared following the PRISMA guidelines. Subgroup analysis and sensitivity analysis were not applicable for this narrative systhesis.

### Assessment of Heterogeneity

According to the study design of data analysis, the assessment of heterogeneity was not applicable.

## Results

### Literature Selection and Characteristics

**FIGURE 1:**
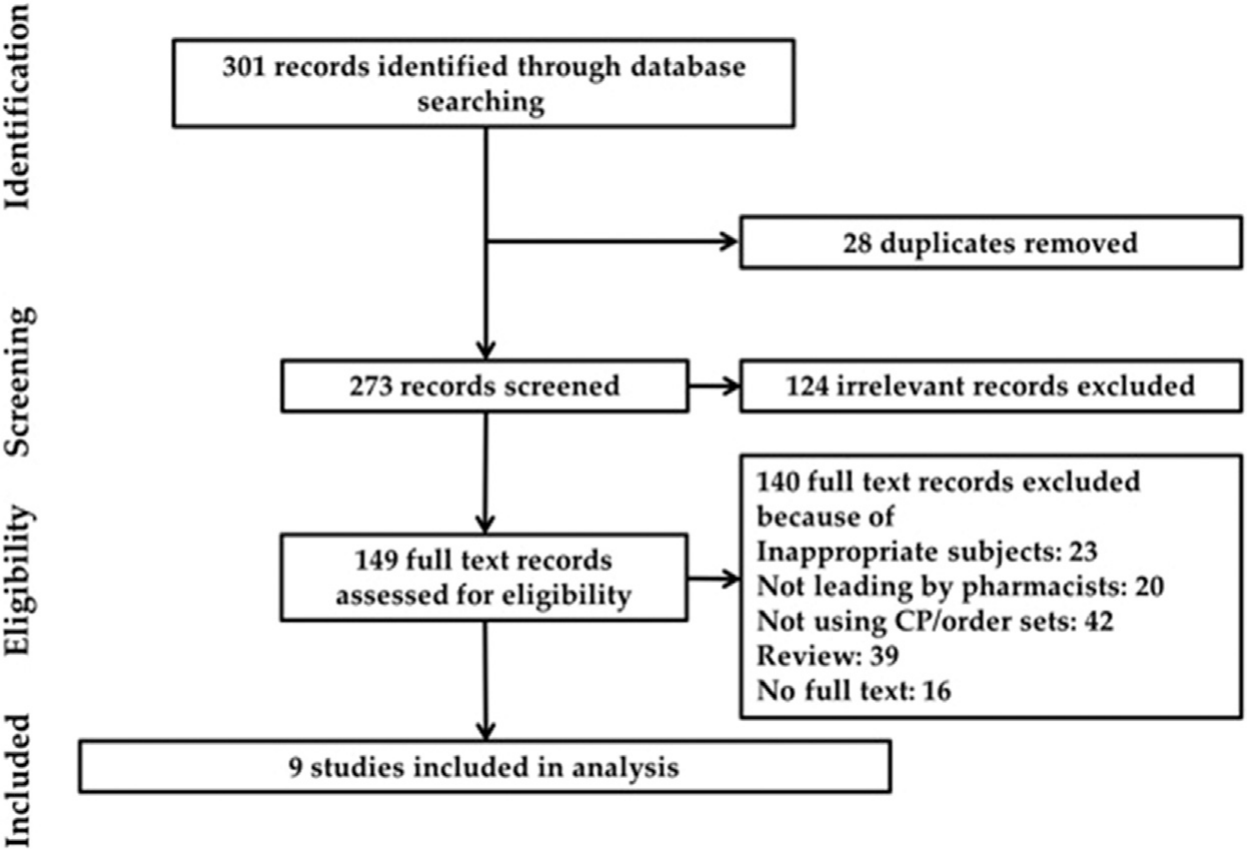
Flowchart illustrating the selection process of studied inclusion.

Initially, the article retrieval of the above databases resulted in 301 records. On screening of the titles and the abstracts, there were 28 duplicates, and 124 irrelevant publications were excluded. After assessing the full texts, 140 were excluded for not meeting the inclusion criteria, three about medication service introduction, 16 conference abstracts, and 39 reviews. The flow process is displayed in Figure 1. Eventually, nine studies were identified to meet the criteria. Among the nine articles, six were UBAs and three case-series studies. The scope of CP/order sets of identified articles can be divided into two types, one focusing on chemotherapy agents (Ise et al. (2003); Nerich et al. (2013); Iwata et al. (2015); Battis et al. (2017); Mcbride et al. (2018))and the other on the managements of chemotherapy-induced complications (Berard and Mahoney, 1995; Best et al., 2011; Hanzelka et al., 2013; Vicente et al., 2016). The characteristics of each study are given in Supplementary Table S1.

### Quality Appraisal

The bias assessment was only performed on six UBAs (Supplementary Table S2). A tool modified from NOS was utilized for the appraisal, transferring the term “exposure” to “intervention.” Four studies had an overall fair quality, which indicated a low risk of bias. Two studies were determined as poor quality, indicating the risk of bias.

### Outcome Classification

Outcomes from included articles are illustrated in Supplementary Table S3. They can be categorized into five themes: economic savings, changes in length of stay (LOS), decreasing antibiotics time intervals of febrile neutropenia (FN), reducing adverse drug reactions (ADRs), and other endpoints.

### Economic Savings

The systematic review found three studies evaluating economic effectiveness. Two studies mentioned the cost-reducing effects on medical expenditure of the drug CP/order sets. Berard and her colleagues designed an ondansetron CP (Berard and Mahoney (1995)) that was well accepted by both adult and pediatric oncologists after one year of application. The daily average costs of ondansetron decreased significantly from $145 to $98, and a total of $204,988 was saved in a year. CP/order sets also showed the capacity to reduce hospitalization expenses (intervention: Y17,554 ± 19,448; control: Y36,636 ± 31,657; *p* < 0.001) and saving inpatient bed days, enhancing access to care, and improving financial metrics. Detailed information on economic outcomes is found in Supplementary Table S4.

### Length of Stay

In total, three studies evaluated length of stay (LOS). Outcomes of study conducted by Hanzelka et al. (2013) showed that the 28-day in-hospital mortality was significantly decreased in the after group when compared with that in the before group (*p* = 0.005). The figures of patients who reached their goal blood pressure (*p* = 0.004) and urine output (*p* = 0.002) within the first 6 h of management were significantly better in the after group. But this study cannot achieve any other significant differences in the other outcome measures. A medication management CP for gastrectomy patients (Ise et al. (2003)) concluded that the average number of LOS among the patients who were offered pharmaceutical care compared with those who were not was dramatically prolonged from 5.4 to 26.1 days (*p* < 0.001). However, this study had a negative outcome. A probable explanation given for this result is that three of post-order set patients had unusually long LOS (50, 60, and 64 days, respectively) because of adverse events of sepsis and severe thrombocytopenia due to their chemotherapy.

### Antibiotic Administration for Patients With Febrile Neutropenia or Sepsis

This systematic review found two studies referring to antibiotic administration of FN or severe sepsis (SS) after chemotherapy. One study (Best et al. (2011)) evaluated the percentage of inpatients who were administered antibiotics within 60 min of first FN-related fever. It turned out to be effective, diminishing the 28-day in-hospital mortality in the group after implementation compared with the pre-intervention group (*p* = 0.005). An obvious decrease in time intervals for the initiation of antibiotics was observed for presentation (*p* = 0.031) and order (*p* = 0.012) to antibiotic administration. The order set usage was 31% in the inpatient unit and 71% in the emergency department. As for sepsis management (Hanzelka et al. (2013)), the establishment of a well-designed sepsis order set/algorithms to improve adherence with the noninvasive elements of early goal-directed therapy (EGDT) for sepsis in cancer patients in the emergency was associated with shrink mortality (*p* = 0.005).

### Adverse Drug Reactions

Two articles contained information on adverse drug reactions (ADRs). The PLCOs were mainly used for ADR monitoring and prevention of oral chemotherapy or outpatient settings. Battis et al. (2017) aimed to monitor the adverse reactions to oral drugs, and a predesigned follow-up was performed with all patients on oral chemotherapy. Consequently, seven patients (10.3%) experienced ADRs. The article suggests that intensive monitoring of patients on oral therapy is crucial to handle ADRs and improve patient adherence and safety. Another study paid attention on afatinib-induced diarrhea (Iwata et al. (2015)), which found that Grade III diarrhea occurred in only 7.1%, showing effectiveness in reducing severities.

### Others

Nerich et al. (2013) evaluated PLCOs on prescription error (PE) prevention. Over 1 year, PE incidence was estimated at 1.5%, and therefore 218 PE were avoided. Battis et al. (2017) established an oral chemotherapy monitoring order set to assess the patient adherence to their treatment regimen; drug reconciliation was followed up from the electronic record. Twelve (17.6%) patients did not refill their oral chemotherapy drugs within the expected time frame.

## Discussion

Pharmaceutical care in cancer patients is still problematic and needs to be more standardized. Single articles of the impact of CP/order sets are varied and conflicted (Ise et al. (2003)), and therefore, there is still no standardized definition of what a “clinical pathway” actually constitutes. This lack of an accepted definition of what constitutes a clinical pathway impacts on capacity to empirically test the evidence base and compromises planning, resourcing, development, and implementation of clinical pathways. A lack of consensus regarding research outcomes is not surprising, given the lack of agreement regarding what defines a clinical pathway.

In this study, nine articles were included. It is suggesting that PLCOs were new and still developing, remaining to be explored and popularized. Our results showed close monitoring and follow-up of patients on oral and injectable chemotherapy is crucial to achieve intended therapeutic outcomes (Best et al. (2011); Battis et al. (2017); Mcbride et al. (2018)), improve adherence to evidence-based practice (Vicente et al. (2016)), and to reduce healthcare costs. With CP or electronic chemotherapy ordering, we may be able to decrease waiting times for chemotherapy and biochemical examinations, which is of considerable significance to cancer patients developing emergent FN (Hanzelka et al., 2013). This suggests that PCLOs may optimize the overall medical efficiency.

The antibiotic administration is a critical issue in cancer patients with FN. As for SS, the in-hospital survival rate for those who received antibiotics within 60 min of the start of hypotension was 79.9% (Kumar et al., 2006). But with the delay of administration, the death rate increased dramatically by about 7.6% per hour over a total time period of 6 h. In addition to increases in mortality, the postponement in appropriate antibiotics after the onset of hypotension also increases the risk ratio of acute kidney injury that could also be closely related to mortality (Bagshaw et al., 2009). Consequently, evidence-based and well-designed PLCOs are beneficial in shortening antibiotic prescription intervals, for a decreased in-hospital mortality and LOS.

No relevant RCTs or standard cohort studies were identified. All publications were UBAs and case-series designs. According to Cochrane Handbook for Systematic Reviews of Interventions (Asaro et al., 2006), the heterogeneity of CBAs and UBAs is higher than that of RCTs. Theory and Practice Of Systematic Review/Meta-analysis edited by Wei WL Yi (2013) et al. recognized RCTs as the “golden standard,” followed by cohort study and case–control study. Before-and-after study designs and case-series designs evaluate the changes in clinical practice rather than to answer a clinical research with a preset goal and evaluation. Thus, UBAs and case-series designs have their inherent limitation in study design, endpoint analysis, and baseline comparison, which may explain the large heterogeneity among included studies.

Although the disease of inclusion for systematic review was cancer, outcomes vary from study to study. These outcomes can be categorized into three types: medical effectiveness optimization, physician adherence, and economic effects. Other detailed outcomes, like time of intravesical catheterization, time to regular diet after surgery, and positive culture rate, were also evaluated, which indicate the variety of work that can be carried in practice.

A lack of statistical significance in the reduction in LOS may not be surprising as the pathway is designed to focus on clinical factors, improving care quality, and reducing variation and adverse events, and not on early discharge. Although no clinical outcomes can be assessed by meta-analysis, other studies led by healthcare professionals have evaluated above outcomes related to the implementation of CPs, reflecting a positive result. One study (Zuckermann et al. (2008)), appraised the adherence with institutional CPs for the management of FN and the impact on various outcomes including primary endpoints. The study recorded a shrink in all-cause mortality after CP implementation (24.4 vs. 14.4%; *p* = 0.017), and partial compliance (67.9%) in most cases was found. Basically, as an emerging and still developing pharmaceutical care pattern, the CP/order sets had positive effects in most studies.

The results indicated that both inpatients and outpatients can be benefited from PLCOs. Establishing PLCOs allowed the assessment of adherence and reeducation on the importance of adherence when deemed necessary (Battis et al., 2017). There was an improvement in time to discover a medication error and provide prompt action for correction for better outcomes in several studies. It will be more interesting to determine the impact of the clinic based on a cost avoidance analysis because of its role in addressing patient adherence and restricting the day supply rule, which is worth being investigated in our future study.

Due to the variations in populations, research design, and outcomes, this systematic review had inherent limitations. The most significant limitation faced was outcome variations, followed by methodology shortage, as well as inter-study inconsistencies (Higgins JPT, 2011). This precluded the authors from performing quantitative analysis for all included research. As a consequence, all reviews performed on this subject are at risk of different types of heterogeneity due to various populations, research designs, and study settings, as well as of unavoidable bias due to nonhomogeneous definitions. Potential limitations of this study could be associated with the appraisal tool for UBAs that still needed to be modified. Evidence identified and included in the study was not high-grade and excluded many conference abstracts. Therefore, more extensive database searching is needed to find higher quality research to evaluate PLCOs in clinical application. Last but not least, the number of included studies was small due to the special topic, which indicated that we need to update the results continuously.

## Conclusion

In summary, this is the first study to evaluate the effect of PLCOs on different clinical outcomes so far. Although the results may be at risk of different types of heterogeneity, the emerging concept of pharmacotherapy CP/order sets has relative benefits in effectiveness and economic endpoints of cancer patients. Nevertheless, more exploration and optimization of PLCOs is needed, for a better and widespread application.

## Data Availability

The raw data supporting the conclusion of this article will be made available by the authors, without undue reservation.
