# Supplementary material for: Effects of Pharmacist-Led Clinical Pathway/Order Sets on Cancer Patients: A Systematic Review
Source: Front Pharmacol. 2021 May 21;12:617678. doi: 10.3389/fphar.2021.617678 (PMC8176097; doi:10.3389/fphar.2021.617678)
Supplement: Supplementary file 1 [file DataSheet1.PDF]

Supplement 1. Search strategy of the manuscript

- **PubMed, limit to human, from inception to March 2020**

(Neoplas* OR Tumor* OR Malignancy OR Malignancies OR Cancer* OR Therioma OR Lymphoma OR Leukemia) AND (“Critical Pathway” OR “Critical Paths” OR “Critical Path” OR “Clinical Paths” OR “Clinical Path” OR “Clinical Pathways” OR “Clinical Pathway” OR “Treatment algorithm” OR “Treatment algorithms” OR “Medical algorithm” OR “Medical algorithms” OR “Order template” OR “Order templates” OR “Order set” OR “Order sets” OR “electronic prescribing”) AND (“Pharmaceutic Services” OR “Pharmaceutic Service” OR “Pharmaceutical Service” OR “Pharmaceutical Services” OR “Pharmacist intervention” OR “Pharmacy Services” OR “Pharmacy Service” OR “Hospital Pharmaceutical Service” OR “Hospital Pharmaceutical Services” OR “Hospital Pharmaceutic Service” OR “Hospital Pharmaceutic Services” OR “Pharmaceutical care” OR Pharmacist OR “clinical pharmacist” OR “Clinical Pharmacists” OR “Pharmacist-driven”)

- **Embase, limit to human and only include standalone Embase database, from inception to March 2020**

(Neoplasia OR Neoplasm OR Tumor OR Tumors OR Malignancy OR Malignancies OR Cancer OR Cancers) AND (‘Critical Paths’ OR ‘Critical Path’ OR ‘Clinical Paths’ OR ‘Clinical Path’ OR ‘Clinical Pathways’ OR ‘Clinical Pathway’ OR ‘Treatment algorithm’ OR ‘order template’ OR ‘Order set’ OR ‘Care pathway’)

- **Cochrane Library, from inception to March 2020**

(Neoplasia OR Neoplasm OR Tumor OR Malignancy OR Malignancies OR Cancer OR Cancers) AND (“Critical Paths” OR “Critical Path” OR “Clinical Paths” OR “Clinical Path” OR “Clinical Pathways” OR “Clinical Pathway” OR “Treatment algorithm” OR “order template” OR “Order set” OR “Care pathway”)

- **Clinical trials and other grey literatures, from inception to March 2020**

(Neoplasia OR Neoplasm OR Tumor OR Malignancy OR Malignancies OR Cancer OR Cancers)

AND (“Critical Paths” OR “Critical Path” OR “Clinical Paths” OR “Clinical Path” OR “Clinical Pathways” OR “Clinical Pathway” OR “Treatment algorithm” OR “order template” OR “Order set” OR “Care pathway”)
