# Supplementary material for: Effects of Pharmacist-Led Clinical Pathway/Order Sets on Cancer Patients: A Systematic Review
Source: Front Pharmacol. 2021 May 21;12:617678. doi: 10.3389/fphar.2021.617678 (PMC8176097; doi:10.3389/fphar.2021.617678)
Supplement: Supplementary file 2 [file DataSheet2.docx]

**Tables**

**Table 1. The concrete characteristics of included studies**

| **Article** | **Research Type** | **Region** | **Subject** | **Intervention**  **（n=）** | **Control**  **（n=）** | **Outcomes** | **Intervention Type** |
| --- | --- | --- | --- | --- | --- | --- | --- |
| Battis, B 2016 | Case-series | US | Outpatient oral chemotherapy | 71 | NR | D, E | Order Set |
| Iwata, K 2015 | Case-series | Japan | NSCLC patients using Afatinib | 14 | NR | D | CP |
| Nerich, V 2013 | Case-series | France | Cancer patients in clinic | 218 | NR | A, E | Order Set |
| Mildred Vicente 2017 | UBA | US | Cancer patients with FN | 131 | 236 | C | CP |
| Ali McBride 2018 | UBA | US | Transition of chemotherapy to the outpatient | 75/192 | 27/39 | A, B | Order Set |
| Katy M. Hanzelka 2013 | UBA | US | Cancer patients with sepsis | 100 | 100 | B | CP |
| Janie T. Best 2011 | UBA | US | Heamatological malignancies with FN | 23 | 30 | C | CP |
| Berard, C. M 1995 | UBA | US | Chemotherapy induced nausea and vommiting | NR | NR | A | TA |
| Ise, Y 2003 | UBA | Japan | Gastric patients with gastrectomy | 46 | 36 | A, B | CP |

（*CP=Clinical Pathway; TA=Treatment Algorithms; FN=Febrile Neutropenia）

A: economic endponts; B: LOS; C: FN; D: ADR; E: others

**Table 2. Quality appraisal of UBAs with full texts**

| **Study** | **Mildred Vicente 2017** | **Ali McBride 2018** | **Katy M. Hanzelka 2013** | **Janie T. Best 2011** | **Berard, C. M 1995** | **Ise, Y 2003** |
| --- | --- | --- | --- | --- | --- | --- |
| **Selection** |  |  |  |  |  |  |
| Representativeness of the after-intervention cohort | 1 | 1 | 1 | 1 | 1 | 1 |
| Selection of the before-intervention cohort | 1 | 1 | 1 | 1 | 0 | 1 |
| Ascertainment of intervention | 0 | 1 | 1 | 0 | 0 | 1 |
| Demonstration that outcome of interest was not present at start of study | 1 | 1 | 1 | 1 | 1 | 1 |
| **Comparability** |  |  |  |  |  |  |
| Comparability of cohorts on the basis of the design or analysis | 1 | 1 | 2 | 1 | 0 | 1 |
| **Outcome** |  |  |  |  |  |  |
| Assessment of outcome | 1 | 0 | 1 | 1 | 1 | 1 |
| Was follow-up long enough for outcomes to occur | 1 | 1 | 0 | 0 | 0 | 0 |
| Adequacy of follow up of cohorts | 0 | 0 | 0 | 0 | 0 | 0 |
| **Total** | 6 | 6 | 7 | 5 | 3 | 6 |
| **Overall Quality** | **Fair** | **Fair** | **Fair** | **Poor** | **Poor** | **Fair** |

**Table 3. Outcomes in included studies**

| **Article** | **Economic endpoints** | **Length of Stay** | **Febrile Neutropenia** | **ADR** | **Others** |
| --- | --- | --- | --- | --- | --- |
| Battis, B 2016 |  |  |  | √ | √ |
| Iwata, K 2015 |  |  |  | √ |  |
| Nerich, V 2013 | √ |  |  |  | √ |
| Mildred Vicente 2017 |  |  | √ |  |  |
| Ali McBride 2018 | √ | √ |  |  |  |
| Katy M. Hanzelka 2013 |  | √ |  |  |  |
| Janie T. Best 2011 |  |  | √ |  |  |
| Berard, C. M 1995 | √ |  |  |  |  |
| Ise, Y 2003 | √ | √ |  |  |  |

**Table 4. Detailed information on economic outcomes**

| **Research Name** | **Study Intervention** | **Endpoints** | **Results** | **Conclusion** |
| --- | --- | --- | --- | --- |
|  |  |  |  |  |
| Berard, C. M.1995 | TA | Daily ondensetron expense Cost avoidence | Intervention: $98; Control: $145 $204988 | An antiemetic treatment algorithm, integrated with a preprinted physician order form, was well accepted and has reduced expenses for antemetic therapy |
| Ise, Y2003 | CP | LOS, days Medication dispense, JPY | Intervention Control P 26.1 ± 3.8 5.4 ± 6.6 ＜0.001 Y17,554 ± 19,448 Y36,636 ± 31,657 ＜0.001 | A CP employing medication management and instruction tasks for gastrectomy patients may play a substantial role in saving on medical costs. |
| Ali McBride 2018 | Order Sets | Inpatients bed days Cost-saving | Saving 747 inpatient bed days Saving costs $1,402,866; Cummulative cost saving $3,305,756 | Enhanced access to care, decreased bed utilization in the hospital, and improved clinical and financial metrics |

**Table 5. Detailed information about the study involving LOS**

| **Research Name** | **Study Intervention** | **Endpoints** | **Results** | **Conclusion** |
| --- | --- | --- | --- | --- |
|  |  |  |  |  |
| Katy M. Hanzelka2013 | CP | 28-day in-hospital mortality, n% LOS, days ICU length of stay, days Goal urine output, n% Goal blood pressure, n% Time to vasopressor, minutes Positive cultures, n (%) Time to first antibiotic, minutes | Intervention Control P 20(20%) 38(38%) 0.005 8.1±6 10.3 ± 11.6 0.160 2.5 ± 3.3 5.1 ± 10.0 0.067 70 (96 %) 55 (79 %) 0.002 90 (90 %) 74 (74 %) 0.004 94 ± 139 138 ± 177 0.054  52 (52 %) 61 (61 %) 0.199 103 ± 103 114 ± 101 0.432 | Implementation of a standardized sepsis order set was associated with a decreased 28-day in-hospital mortality rate. |
| Ise, Y2003 | CP | LOS, days Medication dispense, JPY | Intervention Control P 26.1 ± 3.8 5.4 ± 6.6 ＜0.001 Y17,554 ± 19,448 Y36,636 ± 31,657 ＜0.001 | CP may play a substantial role in saving on medical costs. |
| Janie T. Best 2011 | CP | Order to administration, minute LOS, day | Intervention Control P 80.5 ± 78.37 161.64 ± 128.48 0.012  17.43 11.33 ＜0.05 | The order set antibiotics helped reduce time intervals for initial antibiotic therapy |
